# Addressing Oral Health Disparities in Pregnancy: Oral Health Risk Factors and Clinical Findings at a Safety-Net Hospital in the Bronx

**DOI:** 10.1089/whr.2024.0187

**Published:** 2025-03-27

**Authors:** Megan Cloidt, Parth Shah, Erica Robles, Molly Findley, Nadia Laniado

**Affiliations:** ^1^ Department of Dentistry/OMFS, Jacobi Medical Center, Bronx, New York, USA.; ^2^ Department of Dentistry, Albert Einstein College of Medicine, Bronx, New York, USA.; ^3^ Department of Obstetrics & Gynecology, Jacobi Medical Center, Bronx, New York, USA.; ^4^ Department of Obstetrics & Gynecology, Albert Einstein College of Medicine, Bronx, New York, USA.

**Keywords:** access to health care, dental care, oral health, pregnancy, prenatal care, social determinants of health

## Abstract

**Background:**

Pregnant women have been identified as a special adult population that is particularly vulnerable to oral diseases. The aims of this study were (1) to determine the prevalence of oral disease risk factors and (2) to examine the association between having a dental visit in the last 12 months and obvious tooth decay among a sample of pregnant women with low income.

**Methods:**

This cross-sectional study analyzed the outcomes of oral health risk assessments for 554 pregnant women in a municipal hospital in the Bronx, New York. Descriptive statistical analyses were performed to examine the characteristics of the study population. Simple and multivariable logistic regression analyses were conducted to examine the association between having a dental visit in the last 12 months and obvious tooth decay.

**Results:**

Overall, 28.2% of participants had a dental visit in the last 12 months and 87.7% had dental insurance. Over half of individuals reported frequent consumption of sugary beverages (52.2%). Nearly 30% of the participants showed signs of obvious tooth decay. There was no statistically significant association between last dental visit and obvious tooth decay (odds ratio = 1.02, 95% confidence interval [0.67–1.56]).

**Conclusions:**

The high prevalence of unmet oral health needs despite widespread dental insurance coverage in this study sample suggests other barriers to oral health care beyond insurance coverage. The findings from this study underscore the complexity of oral disease and the potential role that targeted, interprofessional efforts can have on the promotion of oral health in vulnerable pregnant women.

## Introduction

Pregnant women have been identified as a special adult population that is particularly vulnerable to oral disease, specifically dental caries and periodontal disease.^1–3^ Studies have demonstrated a high burden of dental problems among the pregnant population and in women of childbearing age in the United States.^4–6^ Socioeconomic factors such as race and ethnicity, household income, level of education, insurance status, and level of social support impact oral health issues for pregnant women.^5,7,8^ The crisis of poor oral health during pregnancy has been highlighted by influential government agencies and health organizations, such as the National Institutes of Health,^9^ the American College of Obstetricians and Gynecologists,^10^ and the American Public Health Association.^1^

Changes in the oral cavity during pregnancy can occur due to physiological changes, such as hormonal fluctuations, and/or behavioral changes, such as frequent vomiting and increased snacking.^1,2^ Importantly, oral disease has been shown to be associated with adverse pregnancy outcomes. Periodontitis, an oral inflammatory disease that causes destruction of the alveolar bone and periodontal ligaments that anchor the teeth, is associated with an increased risk of gestational diabetes mellitus,^11^ preeclampsia,^12,13^ and delivering preterm^13–15^ and/or low birth weight babies.^13,16^ This association may be influenced by a bidirectional inflammatory relationship between periodontal microbiota and increased levels of estrogen and progesterone in the plasma during pregnancy.^2^ Dental caries, a chronic disease caused by bacteria that use sugar to create an acidic erosion of the teeth, has been less studied in terms of association with birth outcomes. However, it is well established that severe dental caries can impact quality of life,^17,18^ lead to interruptions in daily responsibilities such as work and school,^19,20^ and can even lead to acute life-threatening systemic infection.^21^

Addressing the oral health of mothers during pregnancy is an example of primordial prevention, which precedes primary prevention by avoiding the development of a risk factor.^22^ In addition to the proximal health risks that poor oral health can pose to a pregnant mother and her developing fetus, the mother’s oral health status, knowledge, and beliefs may influence the oral health of her children.^23–25^ Multiple studies have demonstrated an association between maternal levels of harmful oral bacteria, such as *Streptococcus mutans*, and early childhood caries.^23,24^ Delivering oral health care during pregnancy has been shown to reduce oral health problems and takes advantage of the opportune “teachable moment” when women may be motivated to adopt health behaviors, as well as a special window for Medicaid insurance eligibility with dental benefits across the United States.^1,26^

The population of interest in this study included pregnant women who sought prenatal care at Jacobi Medical Center, a municipal safety net hospital located in Bronx County, New York. The aims of this study were to (1) determine the prevalence of risk factors as well as clinical signs and symptoms of oral disease and (2) examine the association between having a dental visit in the last 12 months and obvious tooth decay.

## Methods

### Study design and participants

This cross-sectional study utilized deidentified patient data that were previously collected as part of clinical services in the Jacobi Medical Center Department of Women’s Health Services from November 2020 to December 2022. The population of interest in this study included pregnant women who sought prenatal care at Jacobi Medical Center, a municipal safety net hospital located in Bronx County, New York. Jacobi Medical Center is part of the New York City Health and Hospitals Corporation, the largest municipal hospital system in the country, which contains 11 safety-net hospitals. Jacobi Medical Center serves a racially and ethnically diverse lower-income population including, but not limited to, individuals of African American, Hispanic/Latino, South Asian, East Asian, Middle Eastern, and European descent.

The Bronx, the northernmost borough of NYC with 1.4 million residents (17% of the city population), has the highest proportion of non-White residents: 44% Black/African American, 57% Hispanic/Latino, and 34% foreign-born.^27^ It has the state’s lowest educational attainment, highest household poverty, and worst health outcomes, including high chronic disease rates.^27^ Designated as a Dental Health Professional Shortage Area, the Bronx faces significant socioeconomic and health challenges.^27,28^

### Study procedures and data collection

In an effort to expand and enhance interprofessional collaboration with the hospital’s Department of Women’s Health Services, general practice dentistry residents collaborated with Obstetrician/Gynecologists (OBGYNs) and Certified Nurse Midwives (CNMs) to conduct oral health risk assessments during prenatal medical appointments. Oral health risk assessments were conducted by dental providers (specifically, general practice dental residents) in the Department of Women’s Health Services over 2 years (2020–2022) utilizing a screening instrument titled “Adult Oral Health Screening and Risk Assessment.” The screening instrument was adapted from a pediatric version utilized in a prior study that focused on the hospital’s pediatric population.^25^ The assessments consisted of two parts: (1) a questionnaire and (2) a limited oral examination. The questionnaire was administered verbally from the dental provider to the patient, and a medical translator was used if the patient was not proficient in English or if they preferred to use one. The oral examination was conducted by the dental provider, who used light (either from dental loupes or handheld penlight), a disposable dental mirror, and gauze.

The oral health risk assessment data were collected on paper forms during patient encounters and later entered into a secure Microsoft Excel database. The database was only accessible on password-protected hospital computers by study personnel within the Department of Dentistry. After data entry, paper forms were destroyed via shredding. Data were then deidentified by removing all personal identifiers before sending the spreadsheet via an encrypted email on the secure hospital server to the biostatistician for analysis.

#### Predictor variable

The main predictor variable was “had a dental visit in last 12 months,” which was defined dichotomously as whether or not the patient had visited a dental provider for any reason in the last 12 months. This variable was captured by the dental provider asking the patient the following question verbally: “Have you had a dental visit in the last 12 months?” The answer choices were “yes” or “no.”

#### Outcome variable

The main outcome variable was “obvious tooth decay,” which was defined as severe decalcification or cavitation of the enamel and/or dentin that could be confidently identified visually without the need to feel tactically for textural change or the use of radiographs. This variable was captured in the clinical dental exam by the dental provider and was defined dichotomously as either having obvious tooth decay or not having obvious tooth decay, regardless of the number of teeth affected.

#### Covariates

The selection of covariates was based on *a priori* knowledge consistent with published studies related to the oral health of pregnant women. The covariates included:

##### Demographics (self-reported)

Age in years, race or ethnicity (non-Hispanic White, non-Hispanic Black/African American, Hispanic, Asian, more than one race, other), and pregnancy trimester (first, second, or third).

##### Risk factors (self-reported)

Dental insurance status (yes/no), brushing frequency (daily, occasionally, never), flossing frequency (daily, occasionally, never), smoking (non-smoker, current smoker, former smoker), frequent snacking between meals (yes/no), and frequent consumption of sugary beverages (yes/no).

##### Symptoms (self-reported)

Pain from the mouth (yes/no), bleeding from the mouth (yes/no), and dry mouth (yes/no).

##### Clinical findings

Loose or broken teeth (yes/no), visible plaque/calculus (yes/no), missing teeth excluding third molars (yes/no), gingival recession (yes/no), gingival inflammation (yes/no), and tooth erosion/wear (yes/no).

### Statistical analysis

Descriptive statistical analyses were conducted to examine the overall characteristics of the study population as well as by having had a dental visit in the last 12 months. Frequencies and proportions were reported for the categorical variables, and means and standard errors were computed for the continuous variables. Chi-squared test was employed to assess the association of each categorical variable with having a dental visit in the last 12 months, and Student’s *t*-test was used to determine the association for continuous variables across having a dental visit in the last 12 months.

Simple and multiple logistic regression analyses were conducted to examine the association of self-reported dental visit in the last 12 months and obvious dental decay. Unadjusted and adjusted odds ratios (OR) and respective 95% confidence intervals (CI) were computed to determine the direction and the magnitude of these associations. The final regression model was controlled for age, race or ethnicity, dental insurance, brushing frequency, flossing frequency, smoking, frequent snacking, sugary beverages, reported dry mouth, and trimester.

All data management procedures and statistical analyses were performed in SAS 9.4 for Windows (SAS Institute Inc., Cary, NC, 2018). Significance level (alpha) was set to 5%, and all hypothesis tests were two sided. This study was approved by the Albert Einstein College of Medicine’s Institutional Review Board (IRB #2023-15392).

## Results

Table 1 presents the characteristics of the study sample overall and by dental visit in the last 12 months. The study sample included 554 adult pregnant women ages 18–55 years, with a mean age of 29.8 (standard error [SE] 0.26) years. Individuals were seen at all stages of pregnancy, with 13.9% in the first trimester, 39.7% in the second trimester, and 46.4% in the third trimester. The race/ethnicity of the majority of individuals was Non-Hispanic Black/African American (32.9%) and Hispanic/Latino (38.1%).

**Table 1.** Characteristics of the Study Sample, Including Pregnant Women 18–55 Years Old, *N* = 554

	Overall	Had a dental visit in last 12 months	Did not have a dental visit in last 12 months	
Characteristics	*N* (%)	*N* (%)	*N* (%)	*p*-Value^a^
	554 (100.00)	156 (28.16)	398 (71.84)	
Demographics				
Age in years^b^	29.8 (0.26)	30.13 (0.47)	29.67 (0.31)	0.42
Race/Ethnicity				
Non-Hispanic White	15 (2.71)	5 (3.21)	10 (2.52)	0.93
Non-Hispanic Black/African American	182 (32.85)	52 (33.33)	130 (32.66)	
Hispanic/Latino	211 (38.09)	56 (35.9)	155 (38.94)	
Asian	54 (9.75)	14 (8.97)	40 (10.05)	
More than one race	38 (6.86)	11 (7.05)	27 (6.78)	
Other	54 (9.75)	18 (11.54)	36 (9.05)	
Pregnancy trimester				
First	77 (13.9)	18 (11.54)	59 (14.83)	0.31
Second	220 (39.71)	58 (37.18)	162 (40.7)	
Third	257 (46.39)	80 (51.28)	177 (44.47)	
Risk factors				
Dental insurance				
Yes	486 (87.73)	140 (89.74)	346 (86.93)	0.36
No	68 (12.27)	16 (10.26)	52 (13.07)	
Brushing frequency				
Daily	529 (95.49)	151 (96.79)	378 (94.97)	0.59
Occasionally	24 (4.33)	5 (3.21)	19 (4.78)	
Never	1 (0.18)	0 (0)	1 (0.25)	
Flossing frequency				
Daily	100 (18.05)	35 (22.44)	65 (16.33)	0.04
Occasionally	210 (37.91)	65 (41.67)	145 (36.43)	
Never	244 (44.04)	56 (35.9)	188 (47.24)	
Smoking				
Non-smoker	436 (78.7)	126 (80.77)	310 (77.89)	0.68
Current smoker	13 (2.35)	4 (2.56)	9 (2.26)	
Former smoker	105 (18.95)	26 (16.67)	79 (19.85)	
Frequent snacking in between meals				
Yes	305 (55.45)	86 (55.84)	219 (55.3)	0.91
No	245 (44.55)	68 (44.16)	177 (44.7)	
Frequent consumption of sugary beverages				
Yes	287 (52.18)	75 (48.7)	212 (53.54)	0.31
No	263 (47.82)	79 (51.3)	184 (46.46)	
Symptoms				
Pain in mouth				
Yes	144 (25.99)	40 (25.64)	104 (26.13)	0.91
No	410 (74.01)	116 (74.36)	294 (73.87)	
Bleeding from mouth				
Yes	274 (49.46)	73 (46.79)	201 (50.5)	0.43
No	280 (50.54)	83 (53.21)	197 (49.5)	
Dry mouth				
Yes	143 (25.81)	31 (19.87)	112 (28.14)	0.05
No	411 (74.19)	125 (80.13)	286 (71.86)	
Clinical findings				
Obvious tooth decay				
Yes	163 (29.42)	44 (28.21)	119 (29.9)	0.69
No	391 (70.58)	112 (71.79)	279 (70.1)	
Loose or broken teeth				
Yes	91 (16.43)	31 (19.87)	60 (15.08)	0.17
No	463 (83.57)	125 (80.13)	338 (84.92)	
Visible plaque/calculus				
Yes	415 (74.91)	109 (69.87)	306 (76.88)	0.09
No	139 (25.09)	47 (30.13)	92 (23.12)	
Missing teeth (excluding third molars)				
Yes	150 (27.08)	48 (30.77)	102 (25.63)	0.22
No	404 (72.92)	108 (69.23)	296 (74.37)	
Gingival recession				
Yes	83 (14.98)	18 (11.54)	65 (16.33)	0.16
No	471 (85.02)	138 (88.46)	333 (83.67)	
Gingival inflammation				
Yes	230 (41.52)	68 (43.59)	162 (40.7)	0.54
No	324 (58.48)	88 (56.41)	236 (59.3)	
Tooth erosion/wear				
Yes	40 (7.22)	12 (7.69)	28 (7.04)	0.79
No	514 (92.78)	144 (92.31)	370 (92.96)	

^a^ Chi-square tests for categorical variables and Student’s *t*-tests for continuous variables were used to assess the association with last dental visit.

^b^ Mean (SE).

SE, standard error.

Table 1 was stratified by last dental visit. Overall, 28.2% of individuals had a dental visit in the last 12 months and 71.8% did not have a dental visit in the last 12 months. There were no statistically significant differences observed between groups across variables with the exception of flossing frequency. The large majority of individuals had dental insurance (87.7%). Of the 71.8% of individuals who did not have a dental visit in the last 12 months, 86.9% reported having dental insurance. When asked about oral hygiene practices, 95.5% of individuals reported daily tooth brushing, while 18.1% reported daily flossing. The majority of individuals were non-smokers (78.7%), followed by former smokers (19%) and current smokers (2.4%). When asked about dietary habits, approximately half of individuals reported frequent snacking between meals (55.5%) and frequent consumption of sugary beverages (51.2%).

When asked questions about symptoms of oral disease, approximately one quarter of individuals (26%) reported pain in their mouth, half (49.5%) reported bleeding in their mouth, and a quarter (25.8%) reported dry mouth. When examined clinically, the measures with the highest prevalence included obvious tooth decay (29.4%) and missing teeth excluding third molars (27.1%). In terms of the gingiva and oral mucosa, three quarters had visible plaque and calculus (74.9%) and 41.5% exhibited gingival inflammation.

Table 2 presents the multivariable logistic regression analyses for the association between last dental visit and obvious tooth decay. There was no statistically significant association found between last dental visit and obvious tooth decay (OR = 1.02, 95% CI: [0.67, 1.56]).

**Table 2.** Association Between Having a Dental Visit Within the Last 12 Months and Obvious Tooth Decay, Multivariable Logistic Regression

	Odds ratio (95% CI)	*p*-Value
Model 1^a^ (reference = yes)	1.09 (0.72–1.64)	0.69
Model 2^b^ (reference = yes)	1.02 (0.67–1.56)	0.93

^a^ Model 1—Unadjusted model, N = 554.

^b^ Model 2—Adjusted for age, race or ethnicity, dental insurance, brushing frequency, flossing frequency, smoking, frequent snacking, sugary beverages, dry mouth, and trimester. N = 550.

CI, confidence interval.

## Discussion

This study highlights the significant oral health challenges faced by pregnant individuals receiving care at a safety-net hospital in the Bronx, despite nearly universal dental insurance coverage in this population. Approximately 72% of participants did not have a dental visit in the past year, and nearly one third presented with obvious tooth decay. These findings reveal a critical unmet need for dental care, further compounded by the lack of a significant association between recent dental visits and the resolution of untreated dental decay.

These findings underscore three major areas of concern and opportunities for future research: (1) to better understand the behavioral and clinical risk factors contributing to oral health issues in this population, (2) to identify underlying barriers to accessing dental care and completion of treatment, and (3) to identify effective interventions to improve this population’s oral health outcomes. These identified areas will guide our future research and program planning agenda, as we seek to address the multifactorial challenges underlying oral health disparities in this vulnerable group.

### Behavioral and clinical risk factors

While most participants reported protective behaviors such as daily tooth brushing and abstaining from smoking, frequent sugary beverage consumption was a notable risk factor, affecting 52.2%, which was substantially higher than the 21.9% reported in U.S. pregnant women via Behavioral Risk Factor Surveillance System data.^29^ Sugar-sweetened beverages (SSBs) contribute to a multitude of health conditions such as type 2 diabetes, obesity, and hypertension, making this an issue that warrants public health concern and requires interprofessional collaboration to address. Consumption of SSBs has been highlighted by the American Dental Association as a key area of public health concern and has been associated with increased dental caries experience in both children and adults.^30^ In the pregnant population, SSBs have been shown to be associated with increased rates of weight gain,^31,32^ gestational diabetes,^31,33,34^ preeclampsia,^31,34^ and preterm delivery.^31,34–36^

In terms of clinical risk factors, obvious tooth decay was found in almost one third of this population (29.4%), exceeding the 21.3% prevalence of untreated tooth decay in the general U.S. adult population, according to the National Health and Nutrition Examination Survey.^37^ Untreated tooth decay can lead to pain and infection, which could be particularly dangerous in pregnant individuals. There was also a high prevalence of individuals reporting pain (26%) and bleeding in their mouths (49.5%). Pain in the mouth could be caused by many factors, including but not limited to dental caries. Bleeding is often a clinical sign of gingival inflammation (i.e., gingivitis), which is reversible and may or may not represent current or future periodontal disease.

### Barriers to dental care utilization

Despite almost 90% of the sample reporting dental insurance coverage, only 28% of participants visited a dentist within the last year, a utilization rate far below the approximate 45% utilization rate for preventive dental visits for pregnant individuals in states with Medicaid dental benefits.^38^ This finding suggests that barriers beyond insurance coverage are driving poor dental utilization. These barriers likely include difficulty finding dental providers who accept Medicaid, socioeconomic constraints (e.g., transportation, childcare, out-of-pocket costs),^39,40^ and provider reluctance to treat patients who are pregnant due to knowledge gaps, fear, and/or liability concerns.^41–43^ Low oral health literacy further exacerbates these challenges, as many individuals who are pregnant remain unaware of Medicaid dental benefits and the importance of oral health during pregnancy.^40,44^ For example, a Maryland study found that most pregnant women with low income lacked understanding of caries prevention and Medicaid dental benefits, highlighting the critical need for educational interventions in this population.^44^

### Effective interventions

Novel oral health-focused interventions are underway at our institution, as a direct response to the research presented in this article. A new, stand-alone dental operatory was constructed within the Department of Women’s Health and staffed with dental providers including dentists and a dental hygienist. All dental providers were trained to educate and treat pregnant individuals. Additionally, a new referral system was created utilizing our hospital’s shared electronic health record. Oftentimes, women’s health care providers come to speak directly to the dental providers about patients and referrals, highlighting the importance of physical co-location of services in fostering easy interprofessional communication.

Since the program’s inception in 2023, approximately 800 pregnant individuals have received a preventive dental visit and/or disease-focused dental treatment. This intervention was supported not only by this study but also by a growing body of evidence that supports the efficacy of medical-dental integration for the pregnant population.^45,46^

### Strengths and limitations

This study has several limitations. Using a convenience sample limits generalizability, though our findings may apply to similar urban pregnant populations with low income in the United States. The lack of association between having a dental visit in the last 12 months and obvious tooth decay may be due to low statistical power, small sample size, and potential selection bias from using a convenience sample. Additionally, self-reported data surrounding health behaviors during pregnancy could be impacted by recall bias and social desirability bias.

Dental practitioners conducting oral health assessments received similar training in the same hospital residency program but were not specifically calibrated to perform the oral health risk assessments. The study did not include measurements of clinical attachment loss or radiographs, essential for diagnosing periodontal disease, and, therefore, periodontal disease status was not included. Consequently, while we identified self-reported symptoms such as pain and bleeding, clinical details of their etiology remain unclear. Our limited oral examinations likely underestimated the prevalence of caries, missing non-cavitated lesions and interproximal caries, suggesting the actual level of untreated dental caries might be higher than reported.

Despite these limitations, this study has several strengths. To our knowledge, this was the first study to examine the oral health of pregnant individuals in the Bronx, one of New York State’s most vulnerable pregnant populations. Clinical assessments conducted by dentists provided objective measures of oral health, complementing self-reported data. Moreover, the study addressed a broad range of oral health symptoms, enabling a more comprehensive understanding of the population’s needs.

### Implications for practice and research

Our findings underscore the need for a comprehensive, interprofessional approach to oral healthcare for pregnant individuals, involving collaboration between dental providers, primary care physicians, obstetrician-gynecologists, nurse midwives, and other health care professionals.^47^ Interprofessional care models have been shown to increase access to oral health services during pregnancy and offer a cost-effective, sustainable health care delivery system.^48^ Several studies have demonstrated the benefits of integrating oral health care into prenatal medical care, such as increasing dental care utilization among pregnant women.^45,46,49^

Future research should explore barriers and facilitators to accessing dental care in this population and identify other determinants of oral health. Both quantitative and qualitative research methods will be employed to investigate the persistent oral health disparities observed among pregnant individuals in this safety-net hospital setting. This initial study provides critical baseline data that will inform future efforts to improve oral health outcomes and develop targeted interventions tailored to the unique needs of this population.

## Conclusion

Despite widespread dental insurance coverage, pregnant women in this safety-net setting in the Bronx, New York, demonstrate a high burden of unmet oral health needs, dietary risk factors, and low dental utilization. Findings from this study underscore the importance of targeted, interprofessional, and collaborative efforts, with a focus on the social determinants of health, to promote the oral health of vulnerable pregnant women. Future research should focus on continuing to identify barriers and facilitators to accessing oral health care in this population.

## Abbreviations Used

CI: confidence itnerval
CNM: Certified Nurse Midwife
OBGYN: Obstetrician/gynecologist
OR: odds ratio
SE: standard error
SSB: sugar-sweetened beverage
